# Pediatric Tuberculosis Management: A Global Challenge or Breakthrough?

**DOI:** 10.3390/children9081120

**Published:** 2022-07-27

**Authors:** Lehlogonolo N. F. Maphalle, Bozena B. Michniak-Kohn, Modupe O. Ogunrombi, Oluwatoyin A. Adeleke

**Affiliations:** 1 Division of Pharmaceutical Sciences, School of Pharmacy, Sefako Makgatho Health Sciences University, Pretoria 0208, South Africa; francis.maphalle@gmail.com; 2 Ernest Mario School of Pharmacy, Rutgers-The State University of New Jersey, 160 Frelinghuysen Road, Piscataway, NJ 08854, USA; michniak@pharmacy.rutgers.edu; 3 Center for Dermal Research, Rutgers-The State University of New Jersey, 145 Bevier Road, Piscataway, NJ 08854, USA; 4 Department of Clinical Pharmacology and Therapeutics, Sefako Makgatho Health Science University, Pretoria 0208, South Africa; modupe.ogunrombi@smu.ac.za; 5 Department of Pharmaceutical Sciences, College of Pharmacy, Howard University, 2300 4th Street NW, Washington, DC 20059, USA; 6 Laboratory of Parasitic Diseases, National Institute of Allergy and Infectious Diseases, National Institute of Health, Bethesda, MD 20892, USA

**Keywords:** pediatrics, pulmonary tuberculosis, extrapulmonary tuberculosis, diagnosis, treatment, prevention, coronavirus pandemic, COVID-19

## Abstract

Managing pediatric tuberculosis (TB) remains a public health problem requiring urgent and long-lasting solutions as TB is one of the top ten causes of ill health and death in children as well as adolescents universally. Minors are particularly susceptible to this severe illness that can be fatal post-infection or even serve as reservoirs for future disease outbreaks. However, pediatric TB is the least prioritized in most health programs and optimal infection/disease control has been quite neglected for this specialized patient category, as most scientific and clinical research efforts focus on developing novel management strategies for adults. Moreover, the ongoing coronavirus pandemic has meaningfully hindered the gains and progress achieved with TB prophylaxis, therapy, diagnosis, and global eradication goals for all affected persons of varying age bands. Thus, the opening of novel research activities and opportunities that can provide more insight and create new knowledge specifically geared towards managing TB disease in this specialized group will significantly improve their well-being and longevity.

## 1. Introduction

Until the recent emergence of the coronavirus pandemic, a serious threat to global public health and wellness, tuberculosis (TB) was the leading cause of death from a single infectious agent, ranking above infections caused by the Human Immunodeficiency Virus (HIV) and acquired immunodeficiency syndrome (AIDS) [1,2,3]. Even though TB is an ancient disease, it affects people of all ages and gender and remains a major global public health problem to date [2,4,5,6]. TB is an important source of economic devastation, revolving poverty, and illness, which has ensnared families, societies, and even countries globally, with the most vulnerable groups being women, children, and HIV patients [3,6,7]. Since the World Health Organization (WHO) declared the “global tuberculosis emergency” in 2013, a consistent spike in scholarly journals addressing important aspects of the burden, management, diagnosis, and control has occurred, but generally, the emphasis in most cases has been on adult disease. Unexpectedly, pediatric TB has been comparatively neglected, primarily because of the (i) greater challenges associated with its diagnosis and management; (ii) lower priority placed on children by most TB control programs; (iii) considerations around pediatrics being ineffective transmitters of the bacillus; (iv) historical lack of political will and persistent limited availability of required management tools. Thus, research endeavors deliberately focused on pediatrics are noticeably uncommon worldwide [8,9,10,11].

The global burden of pediatric TB remains considerably high, and many cases are seen in children under the age of fifteen. With approximately 1.1 million cases and 230,000 deaths (80% were younger than five years old) reported annually worldwide, plus a much higher risk of severe illness and even death within this fragile group relative to adults; pediatric TB continues to be an ongoing public health challenge, especially in developing countries where there are limited public health infrastructures [6,8,9,10,11,12].

To date, the Bacille Calmette–Guérin (BCG) inoculation is the only vaccine that confers limited protection against TB infection, which often fades away after childhood. Despite the broad diversity in disease pathogenesis and presentation within the pediatric population, TB treatment and prophylaxis are still lengthy, adopt a one-size-fits-all approach, and often contain active drugs that are not yet available in child-friendly dosage forms. Moreover, effective diagnostics are not accessible to pediatric patients and the most reliable, commonly used bacteriological culture approach generally misses about 80% of children with clinically diagnosed infection [10]. Thus, this review discusses the distinguishing features of TB infections within the pediatric community by covering key aspects of prevention, diagnosis, treatment, and, of course, the impact of the ongoing coronavirus pandemic, with particular emphasis on scientific findings over the past decade as well as highlights of important areas that require future investigations.

*Search strategy and selection criteria*: References for this review were identified through searches of PubMed, Google Scholar, and Scopus with the search terms such as “tuberculosis in children and adolescents”, “pediatric tuberculosis”, “tuberculosis treatment or pharmacotherapy”, “tuberculosis diagnostics”, “tuberculosis prevention/prophylaxis in minors” and “coronavirus disease and its impact on pediatric tuberculosis in children and adolescents” majorly over the past decade. Articles were also identified through searches of the authors’ own files. Only papers published in English were reviewed. The final reference list was generated on the basis of originality and relevance to the broad scope of this review manuscript.

## 2. Tuberculosis at a Glance

Tuberculosis (TB) is an infectious disease caused by the bacteria, *Mycobacterium tuberculosis* (*Mtb*). TB is spread through the air in the form of droplet nuclei that are released when an infected person coughs, speaks, or laughs [13]. The droplet nuclei could remain in the air for hours following their release; thus, insufficient ventilation, a lack of ultraviolet light, and minimal aerosolization often accelerate the rate at which the disease is transmitted in an enclosed space [14]. The epidemiology of TB is unevenly distributed, and this is closely related to social determinants such as poverty, increasing populations, and malnutrition. These social determinants influence a person’s vulnerability to TB exposure as well as the potential of individuals to recover from TB infection [15]. Tuberculosis is listed among the top three poverty-related diseases. It is prevalent in areas with poor housing, large and condensed populations, and places where people are highly affected by poverty and malnutrition. The congested accommodations, such as in squatter camps and ghettos, are settings that do not promote adequate cross ventilation and thus aid person-to-person spread. Furthermore, malnutrition, which is common in locations occupied by the poor, results in compromised immune systems; thus, overcrowding, and poor housing will inevitably accelerate the transmission of TB in such settings. Successful control of TB relies on the integration of diagnosis and treatment, as well as the improvement of social determinants. This is evidenced by the relationship between mathematical models and social determinants, which have proved that rates of TB infection in countries offering reduced funding towards TB control systems, in response to a recession, experienced an alarmingly high TB infection rate and significantly reduced case detection [16,17]. The intensity of interventions on proximal factors such as smoking, indoor air pollution, and malnutrition has been known to accelerate the decline in new infections and, consequently, TB incidence [18,19].

Following the coronavirus disease, TB is now the second leading cause of death from a single infectious agent, still ranking above human immunodeficiency virus/acquired immunodeficiency syndrome (HIV/AIDS). In 2020, the WHO reported about 10 million new active infections, and 1.5 million HIV-negative deaths, with an additional 214,000 fatalities among HIV-positive individuals [20]. Tuberculosis is a major global concern and has led to the institution of WHO’s END-TB strategy, which is in line with the United Nation’s sustainable development goals that aim to eliminate the incidence of TB by the year 2035 [21]. A 95% reduction in TB incidence and a 90% decrease in the total number of TB deaths are to be considered as indicators for the satisfaction of the END-TB strategy. Accordingly, the end of TB as a health problem will be best indicated by the incidence rate of fewer than 10 cases/100,000 people, and complete elimination would be defined as having an incidence rate of less than one case/100,000 population [21,22].

Post-*Mtb* transmission and infection, the bacteria may affect the lungs or other organs in the body and therefore result in pulmonary or extrapulmonary TB, respectively. The strength of the host’s immune system determines the severity of the disease that will develop. This can either be latent, active, or miliary TB [13,23]. Latent TB refers to a state of post-infection when *Mtb* is dormant, and non-replicating but triggers persistent immune responses within the host without clinical manifestations or symptoms of active disease [24,25,26]. Active TB develops when the host’s immune system is relatively compromised and has the characteristic symptoms of TB, which include chronic cough with bloody sputum, fever, night sweats, and weight loss [27]. Miliary TB occurs when *Mtb* enters the bloodstream and migrates to other extrapulmonary body parts. It is often rapidly fatal and highly likely to develop when the host’s immune system is severely compromised [27,28].

## 3. Pediatric Tuberculosis

### 3.1. Etiology, Epidemiology, Immunopathology, and Pathophysiology

The incidence recorded in children reflects the failure to control TB in adults [29]. Children are most vulnerable to TB infection because of their immaturity and ultimately impaired immune system [8]. Research has shown that the impaired innate pulmonary defense mechanisms in neonates and children are attributable to the reduced killing of microbes and diminished recruitment of monocytes to the infection site, thus disabling the ability of the immune system to induce necessary responses against the *Mtb* pathogen [8,30]. Furthermore, children are born with ineffective and immature antigen-presenting cells and T helper cells, which disable them from being able to produce interleukin-12 [8,31], which is essential for the initial phase of Type 1 T helper cells (Th1) polarization and for maintaining the efficiency of interferon-γ transcription [32]. This makes children particularly vulnerable to TB infection, accounting for the estimated 10% of all TB cases being those of children under the age of five [27].

About 1.1 million children fall ill with TB annually and approximately 230,000 deaths (including those living with HIV) were recorded in 2020 [2,20,33]. Studies have shown that reported statistics do not provide a true reflection of the global pediatric TB burden because most often, many cases go undiagnosed or unreported and as such, the global statistics could be much higher [6,20,33,34]. It has been observed that most reported pediatric fatalities occur in children who are not receiving any TB treatment, meaning that efforts geared towards identifying groups at risk of infection, effective diagnosis, and appropriate treatment require urgent improvement and implementation to significantly reduce TB mortality in children. Tuberculosis is considered one of the top ten causes of death in children under 15 years, but an important component of the past analysis is the focus on under 5-year-old mortalities. Mathematical estimates of available epidemiological data predicted that TB may be the sixth highest cause of death among children ages one to fifty-nine months, resulting in more mortalities than even meningitis, HIV/AIDS, measles, and pertussis. Moreover, some deaths, often thought to originate from pneumonia, meningitis, or AIDS, may have been caused by TB infection. Thus, some TB-related fatalities were probably not represented in the available global estimates and overall reported numbers are lower than what they really are [35,36,37].

Despite this, pulmonary TB in children remains in the shadows because most incidents are smear-negative and are thus said to have a minor contribution to the spread of the disease [10,38]. Although TB in children is not regarded as a major contributing factor to community spread, it is a key cause of high mortality and morbidity rates among this group largely due to the unlikelihood of early detection, especially in those under 5 years of age, thus accounting for an estimate of over 500,000 new TB cases each year. Most deaths due to active TB infection in pediatric patients are frequently attributed to other diseases such as pneumonia, which is the leading cause of death in children under 5 years [39,40,41]. Although this is underdiagnosed in most cases of pneumonia in children, *Mtb* is a causative agent for both tuberculosis and pneumonia. Literature has reported autopsy results showing that approximately 1–23% of all pneumonia-related cases also presented with TB [40]. This then further supports the notion that pediatric TB cases are underreported. Without a proper estimate of the global TB burden, there would be inadequate allocation of resources for diagnosis, treatment, vaccination, and the market size for potential drug development, which would ultimately motivate pharmaceutical industries to prioritize anti-TB drug development. All these factors are essential tools for the advocacy and control of TB in children [36].

The spectrum of the disease in children ranges from paucibacillary lymphadenitis to severe disseminated disease [23]. Children under 2 years of age, especially fetuses and neonates, are most susceptible to TB infection and are more prone to developing severe disease because their immune systems are underdeveloped. Typically, they have no protection against harmful pro-inflammatory cytokine responses because they are still transitioning from the supposedly sterile intrauterine environment into the antigen-exposed external environment [34,42]. Children less than 5 years are often known not to develop active disease immediately post *Mtb* infection but have a 10% chance of experiencing active disease reactivation during their adulthood [43,44,45]. Moreover, pediatric patients are at greater risk of becoming infected and developing active disease following contact with adult patients who have TB. Children therefore form an important group of patients requiring latent TB infection (LTBI) testing and therapy [23]. Children serve as potential reservoirs for active TB later in their lifetime and this is evidenced by the fact that globally, around 67 million children are carriers of latent TB infection. This poses a significant threat to the success of the World Health Organization’s global “END-TB” strategy [6,27].

### 3.2. Transmission in Children

When children are infected with *Mtb*, they are often more susceptible to getting sick faster than adults who are equally exposed. Relative to the pediatric population, TB disease in adults is typically associated with a previous dormant infection that subsequently develops into an active illness years later, due to immune system weakening often resulting from underlying diseases, drugs, or other environmental factors [46]. In most instances, the transmission of TB amongst pediatrics is because of close and lengthy contact with adults who have infectious pulmonary TB [47,48]. Since children, especially those under the age of two years, are dependent on extensive adult supervision, they will most likely get the TB infection from a close household member, caregiver, or an older child with whom they spend a lot of time. Older children, however, are less dependent on adults and are more social; hence, they usually become infected from an outside source. It can therefore be inferred that transmission in children is not only dependent on social factors such as poverty and overpopulation, but also on the prevalence within communities and the age of the child [42,49,50]. Studies have identified a bimodal risk profile for TB infection in children, which shows that the risk of transmission is highest in those in their late teenage ages and under two years, while the risk decreases significantly for children between five and ten years old [42]. Although it is not considered a major contributing factor toward the spread of infection within the community [39], pediatric TB spread is synonymous with recent infections in societies. The burden of pediatric TB thus provides a more accurate indication of the transmission rates of multidrug-resistant (MDR) and extensively drug-resistant (XDR) strains of *Mycobacterium* [49,51,52]. Children often require approximately twelve months post-infection to progress to an active disease state, which frequently develops into severe forms of extrapulmonary infection such as TB meningitis or miliary TB that may end up being fatal [42,53]. Host-specific factors such as age, history of Bacillus Calmette–Guérin (BCG) vaccination, malnutrition, HIV/AIDS status, etc., can significantly impact the rate and extent of transmission, risk of infection, and progression to active or latent TB [48,54]. 

#### 3.2.1. Active Infection 

##### Pulmonary Infection

Lung-localized infection (pulmonary TB) remains the most common form that occurs in children and symptoms severity are age dependent and present slightly different in every child. Generally, children can have non-specific symptoms that can be mistaken for other conditions leading to a missed diagnosis. Common symptoms in younger children can include fever and chills, weight loss, stunted growth, cough, and swollen glands, while adolescents show slightly different indications such as prolonged cough (over 3 weeks), chest pain, fever and chills, night sweats, appetite reduction, bloody sputum, fatigue and weakness, weight loss, and swollen glands. Testing for pulmonary TB typically involves the skin test and the chest radiography, which show abnormal topography (e.g., presence of airspaces, pleural effusions, cavities, etc.) during active infection [55,56,57,58,59]. 

##### Extrapulmonary Infection

Only about 20–30% of all pediatric cases are extrapulmonary (i.e., spread from lungs to other body parts) in nature and common infection sites include, but are not limited to, lymph nodes, meninges, pleura, miliary dissemination, musculoskeletal system, gastrointestinal tract, kidney, and cutaneous tissue [4,60]. It is noteworthy host–pathogen interactions, which determine the type and severity of extrapulmonary TB that develops in pediatrics, often occur differently within their diverse age groups. For instance, infants are extra prone to more severe forms of extrapulmonary infection such as miliary TB; pre-school-age children suffer more from pleural effusions and TB meningitis, while their older counterparts are more susceptible to skeletal TB [47,61,62,63]. Extrapulmonary TB refers to an indication of *Mtb* through the Ziehl–Neelsen acid-fast strain and culture in Loewenstein–Jensen or BACTEC^TM^ blood culture media in a tissue that was isolated from a body site other than the lung parenchyma. These are often accompanied by imaging studies or clinical findings that are compatible with local TB infection. In negative cultures, extrapulmonary TB is defined as clinical, microbiological, radiographic, or histopathological evidence of *Mtb* anywhere in the body other than the hilar lymph nodes or the lung parenchyma accompanied by a positive tuberculin skin test, history of exposure, and an exclusion of other diseases [64]. Some instances of extrapulmonary TB infection are described below.

1. Extrapulmonary TB with the involvement of the central nervous system:

It only affects about 2% of all TB patients, and 50% of these individuals are children under the age of two. This form of extrapulmonary TB occurs because of hematogenous dissemination of the bacilli from the primary site of infection. The bacilli most typically spread to the meninges, subpial, parenchymal, and subependymal parts of the brain and spinal cord. They can remain inactive for long periods but can also rupture and spread to the cerebrospinal fluid [4,65,66]. The most common and severe form of this disease in pediatrics is TB meningitis [67]. Even with treatment, about 15–40% of affected patients pass away and more than a third suffer from permanent neurological defects after recovery. Patients usually present with febrile illness plus other symptoms such as long-lasting headaches or progressive lethargy that may end up in a coma or even death typically within 5–8 weeks following the onset of the illness [68]. Most cases are diagnosed late, usually at the point when, regardless of the use of appropriate therapy, morbidity and mortality continue to remain high [69,70].

2. Miliary infection:

This refers to the progressive and widespread forms of TB that occur in children because of primary or reactivation of latent infection. The lung, spleen, liver, lymph nodes, meninges, bone marrow, and adrenal glands are frequently affected organs. Symptoms of acute miliary TB in children include septic shock, multiple organ dysfunction syndrome, acute respiratory distress syndrome, and frequent subacute presentation with symptoms progressing to more serious forms such as general unrest. The most used diagnostic tools for miliary TB include chest X-ray, collection of samples from different locations in the body, and biopsy of affected organs for culture and histological testing [68,71,72,73].

3. Lymph node TB:

This is a common form in children and young adults, accounting for approximately 30–40% of all extrapulmonary TB cases. The neck glands are commonly affected, and cervical lymphadenopathy is the most common complication, but the supraclavicular, axillary, thoracic, and abdominal nodes can also be affected [71]. Lymph node TB does not have any systemic involvement; however, necrosis can occur and may produce inflammatory symptoms with ulceration, fistula formation, and scrofula. Disseminated TB infection (miliary TB) can be pulmonary in nature and 18–24% of these are associated with mediastinal affectation. Swelling of the lymph nodes can cause swelling in this location and compress the neighboring structures and cause tracheal, bronchial, or esophageal obstruction [68]. Diagnosis of lymph node TB involves fine-needle aspiration biopsy of the affected lymph nodes, microbiological cytological smear testing, and culture and polymerase chain reaction studies, which are altogether about 77% sensitive and 80% specific for this infection type [74].

4. Skeletal TB:

It is responsible for 20% of pediatric extrapulmonary infections and it most typically occurs in the first twenty years of life, apart from Pott disease (TB of the spine), which normally affects young children. The most common forms of skeletal TB are spondylitis, arthritis, and osteomyelitis [75]. Osteoarticular TB accounts for 11% of all extrapulmonary cases and 50% of these cases present as spondylitis—the most common indication of this type of TB. The lower thoracic regions are commonly affected in younger patients and the upper lumber section in older patients. Localized pain is the most common symptom of this kind of TB [71]. CT scans and X-rays are the tools used to determine the extent of disease progression, affected soft tissues, and degree of neurological involvement. Magnetic resonance imaging is sensitive for assessing neurological involvement [76]. Tuberculosis arthritis typically affects children under eighteen months of age because the transphyseal vessels start to disappear when children grow older [77]. It ordinarily affects the hip and knees and can mimic juvenile idiopathic arthritis [78]. The most common symptoms include swelling, pain, and loss of joint function, which progresses over weeks to months. If diagnosed at a later stage, it presents with joint obstruction, local deformity, and restricted range of movement, while more advanced cases present with fistula formation [71,79].

5. Genitourinary infection:

This involves the infection of the urinary and reproductive system, which develops because of primary or reactivation of dormant TB at this site. It accounts for 14–41% of all extrapulmonary TB scenarios; although, it occurs more in adult males, and approximately 3% of all pediatric cases [80,81,82,83]. Male genital structures usually affected include the prostate, epididymis, and testicles resulting in the development of subacute prostatism and epididymis-orchitis. Incidence in females (~80%) involves the fallopian tubes and causes abdominal pelvic pain and infertility in developing countries [68].

6. Renal involvement:

This initially affects the renal cortex. The bacilli infect the glomerulus and then spread to the corticomedullary junction and medulla. The infection localizes in the kidney parenchyma; hence, the kidney is the most affected organ. Regular symptoms of renal involvement include pyuria and sterile urine [82,84,85].

7. Gastrointestinal and peritoneal TB:

It affects any part of the gastrointestinal tract (GIT) typically including the ileum, jejunum, and colon because these regions are characterized by high-intensity fluid and electrolyte absorption, an abundance of lymphoid tissue, increased physiological stasis, and reduced digestive activity [86,87]. The main etiological agents for gastrointestinal and peritoneal TB are *M. tuberculosis* and *M. bovis,* and this form of disease is not transmittable unless the patient has concomitant pulmonary TB [88]. A person often becomes infected in one of the four following ways: (i) ingestion of milk or food contaminated with *M. bovis,* (ii) swallowing of infected sputum, (iii) hematogenous spread from active pulmonary or miliary TB, or (iv) contiguous spread of the infection from adjacent organs [87]. Abdominal TB accounts for 1–5% of all extrapulmonary incidences in children and it presents with widespread and non-specific symptoms. The most common forms in pediatrics include nodal disease and adhesive peritonitis of either wet or fibrotic type, while extrapulmonary TB with gastrointestinal involvement is most common in adults [86].

8. Cutaneous TB:

It affects the skin, is rare, and accounts for 1–2% of all TB cases [89]. It typically occurs because of the direct spread of *M. tuberculosis* bacilli from infected organs located under the skin, and these are typically the lymph nodes. However, the source of cutaneous TB may also be the synovial fluid, bones, tendons, and joints [90]. It may be paucibacillary or multibacillary in nature depending on the bacilli load from its source, and it may also be endogenous or exogenous based on its route of infection [91,92]. It affects people of all ages, but it is most common in young children, teenagers, and the elderly. This is because individuals in these age groups have weak immune systems, are often malnourished, and can be HIV-positive in some instances [93,94]. Children of ages ten to fourteen years account for a significant proportion of all the cutaneous TB cases, and the infection presents itself with clinical features that resemble those of adults. In the younger pediatric population, cutaneous TB presents with the involvement of the lymph nodes and systemic organs with scrofuloderma being the most common form of cutaneous TB infection in children [95,96]. It is thus of crucial importance that cutaneous infection is diagnosed and treated as early as possible to avoid drastic consequences in children [97]. The diagnosis of cutaneous TB in children is difficult because of its non-specific and variable presentation (i.e., paucibacillary and multibacillary). This usually results in late diagnosis, treatment failures, and the development of drug resistance despite the effectiveness of first-line antitubercular agents in managing this type of infection [98,99]. Treatment failure is further exacerbated by the prevalence of HIV infection, poor living conditions, and missed BCG vaccinations, which account for the rise in cutaneous TB cases in many parts of the world, particularly in India and other Asian countries [97].

##### Drug Resistance 

Active TB infection (pulmonary or extrapulmonary) in pediatrics can be drug-resistant or drug-susceptible (non-resistant). Drug-resistant infection is caused by *Mtb* strains that are insensitive to at least one first-line TB antibiotic. Categories include: (i) Isoniazid resistance (Hr-TB)—which means that the TB bacilli are not sensitive to isoniazid and this constitutes a major impediment to the effective management of TB disease plus latent infection in children; (ii) Rifampicin-resistance (RR-TB)—refers to mycobacterial strains resistant to rifampicin; (iii) Multidrug-resistant TB (MDR-TB)—occurs when the causative bacteria is resistant to more than one TB antibiotic and at least isoniazid and rifampicin; (iv) Extensively drug-resistant TB (XDR-TB)—it is an uncommon kind of MDR-TB in which the *Mtb* strain is resistant to isoniazid and rifampicin, plus any fluoroquinolone and at least one of three injectable second-line drugs (e.g., amikacin, kanamycin, or capreomycin); and (v) the pre-extensively drug-resistant TB, which encompasses resistance to rifampicin with any fluoroquinolone and at least one of the drugs bedaquiline and/or linezolid [2,100,101,102,103].

To date, drug-resistant TB is still a public health crisis and health security menace. It is particularly dangerous because it can be deadly and many young people suffering from the active disease go undiagnosed or misdiagnosed. For instance, the WHO European Region recently estimated that 2000 children under the age of fifteen with drug-resistant TB remain undiagnosed each year and this number excludes adolescents between fifteen and nineteen years old [2,3,104]. In most cases, it emerges when affected persons use TB medicines inappropriately, receive incorrect prescriptions from health care providers, acquire poor-quality drugs, and stop treatment too early. Children and adolescents can also be affected by these factors, but more commonly, they become casualties of drug-resistant TB following primary transmission from direct contact with infected adult patients; thus, the incident rates for these strains in pediatrics serve as indicators of the transmission rates within a community [8,104]. The characteristics of the resistant TB strains and the paucibacillary nature of TB disease in children, lack of adequate diagnostic tests, and limitations associated with record keeping (deaths and new cases) further add to the challenges of dealing with this form of TB infection in pediatric patients [105,106]. 

Treatment of drug-insensitive TB disease is generally protracted (≤eighteen months) and guided by the resistance profile of the infecting *Mtb* strain. To successfully address this, current treatment guidelines involve the two-phased approach (initial and continuation) using second-line medicaments, which typically consist of at least four drugs to which the patient has not been previously exposed. Even though pediatric patients suffering from drug-insensitive TB disease are curable, there is still limited data available on the use of most second-line drugs in pediatrics. This, added to the abovementioned difficulties in diagnosis and therapy of the disease in children, means that there is also a lack of pediatric dosage forms for second-line TB drugs, which leads to decreased patient compliance to treatment, thus adding to the high morbidity and mortality rate associated with drug-resistant infections [8,48,107].

#### 3.2.2. Latent Infection

*Mycobacterium tuberculosis* is a pathogen that primarily causes active TB disease in the lungs but could enter a dormant, non-replicating state that is known as latency or latent TB [25]. The likelihood of a child developing latent TB depends on microbiological, host, and environmental factors. Different strains of *Mtb* elicit varying infection intensities; although, this is not supported by clinical or epidemiological evidence. The closeness of the infected person to the contact case, the duration of interaction, and the infecting individual’s bacterial load plays a crucial role in determining whether a person develops latent or active infection. Children under the age of five years have a higher chance of developing latent TB than those that are older [108,109]. The acquisition of TB infection during childhood contributes to a future reservoir of infectious cases making it even more important [4]. Factors such as age, gender, HIV/AIDS status, and comorbidities such as diabetes mellitus and chronic renal failure alter the immune mechanisms that are responsible for granuloma formation, thereby predisposing children to the activation of latent infection into active disease [71]. The diagnosis of latent TB is challenging because this state of the disease does not display any symptoms and, therefore, infected persons are unsuspecting. It depends on the in vivo or in vitro stimulation of a biological sample with *Mtb* antigens via a tuberculin skin test or interferon-γ-release assays (IGRAs) [109]. The lack of cellulose activity in this dormant state makes it challenging to clear *Mtb* from the infection sites [105,110,111,112]. With the differences in the innate immune responses between adults and children, grown-ups with dormant infection have a 5–15% chance of progressing to active disease during their lifetime, while the probability of this occurring in pediatrics is five times higher particularly when the immune system is compromised [25,109,113,114,115,116]. 

## 4. Management Strategies

### 4.1. Pharmacotherapy

Tuberculosis disease in pediatric patients is treated by taking a combination of antitubercular agents usually for six months for drug-sensitive TB or more if required, particularly when resistance occurs. Regardless of the outcomes of the drug susceptibility testing, TB therapy is primarily focused on patient cure and reduction in *Mtb* community transmission, particularly amongst caregivers [106]. It is imperative to note that TB disease can reoccur in children if drug administration is stopped before completion of the prescribed course. In addition, if drugs are taken incorrectly, the bacteria that are still active may be harder and more expensive to treat, become resistant, and make treatment last much longer (up to eighteen to twenty-four months) [46]. The treatment of drug-susceptible or resistant TB is similar in both adults and children with variations in terms of the dosing and combinations [101,117]. Children tolerate antitubercular drugs much better than adults and are at a lower risk for toxicity. Specific drug regimen selection for pediatric patients is primarily dependent on the identity of the disease-causing *Mtb* strain (i.e., drug-sensitive or resistant). Usually, the regimen is extrapolated from adult doses but because of the pharmacodynamic, physiological, and developmental differences between adults and children, pediatric doses must be adjusted according to the child’s body weight and age [23,118]. 

Inaccurate antibiotic dosing continues to impede desirable pharmacotherapeutic outcomes in pediatric patients suffering from active TB disease. It is expected that the recommended dose of anti-TB drugs in children will lead to a pharmacokinetic profile that generates systemic effects like that of adults exposed to the same drug levels in terms of safety and efficacy [119]. However, parameters of quantifying the optimal dose such as the maximum concentration (C_max_) or the area under the concentration–time curve (AUC) often do not yield reproducible outcomes in adults and children. There is a high correlation between C_max_ and AUC in different adults, but the rapid metabolism/elimination of drugs in children usually results in AUC values usually below that of adults, even if the C_max_ is similar in adults and children. It is for this reason that establishing a pediatric dose targeting adult C_max_ or AUC leads to substantially different doses and therapeutic effects, thus further supporting the need to modify pediatric doses based on the child’s body mass and developmental stage [10,119]. 

Approximately 80% of deaths associated with active pediatric TB infection occur in groups less than five years old, with nearly 90% of these cases made up of children not started on any antitubercular agents [27,28,36]. This reported observation is causally related to the existence of huge gaps between case discovery, treatment initiation, and prophylaxis within this age group. The lack of political involvement, limited allocation of resources, and a substantial global shortage of suitable pediatric dosage forms have further aggravated this disease management flaw and treatment failure rates [10]. Apart from these factors, TB symptoms manifest differently in children of varying age groups, and it is therefore impossible to assume that an all-inclusive treatment approach would yield desirable therapeutic outcomes [4]. The standard drugs for treating drug-susceptible, active TB in children are the same as those for adults. However, in HIV-positive children, therapy is based on distinct guidelines set by the WHO, while there is no difference in the treatment strategies adopted in the management of HIV-positive and -negative children who are infected with drug-resistant *Mtb* strain [6,101]. 

#### 4.1.1. General Classification of Antitubercular Agents

Antitubercular agents being used for the treatment of active drug-sensitive and -resistant infections are generally classified as described below.

##### Group 1 

This category includes the first-line drugs, namely, isoniazid, pyrazinamide, and rifampicin, as core drugs and ethambutol is a companion drug [120,121]. The standard treatment regimen for children (particularly for drug-susceptible infection) includes two months of rifampicin (10–20 mg/kg with a maximum dose of 600 mg/day), 30–40 mg/kg of pyrazinamide, 10–15 mg/day of isoniazid with a maximum dose of 300 mg/day, and, if the benefit outweighs the risk, 15–25 mg/kg of ethambutol. This is then followed by four months of treatment with isoniazid and rifampicin in the abovementioned doses [117,122]. High-dose isoniazid can be included in the MDR/XDR-TB regimen when katG mutation is not documented, but it should not be considered as an active component of the treatment. Though the pyrazinamide susceptibility test is usually undependable, it should still be added for treatment purposes but should not be considered as part of the active drugs [120].

Isoniazid is a primary antitubercular agent. It is bactericidal and acts by rapidly killing actively metabolizing extracellular bacilli through the inhibition of *Mtb* cell wall synthesis, thus providing extended sterilization. Due to its potent bactericidal effect during the intensive phase, it offers protection to other companion drugs included in the treatment [123]. It competes with the pharmacologic and therapeutic effects of vitamin B6 in the biosynthesis of neurotransmitters; thus, supplementation with vitamin B6 is essential [124]. Hepatic and neurologic toxicity in children is rare but can be magnified in malnourished children, those receiving high doses of isoniazid, or who are HIV-positive. The manifestation of these adverse effects is also influenced by the child’s acetylating capacity in which case children regarded as slow acetylators tend to experience more of its toxic effects than fast or intermediate acetylators [125].

Rifampicin possesses both bactericidal and sterilizing activity and acts by inhibiting ribonucleic acid (RNA) synthesis. It kills extracellular and slow-growing intracellular bacilli, thus contributing to its sterilizing activity [123,126]. It is an essential TB antibiotic used in all treatment phases and an inducer of several drug-metabolizing enzymes, especially the cytochrome CYP450 isoenzymes. This leads to a reduction in plasma concentrations and effectiveness of other orally co-administered bioactive that undergo extensive first-pass metabolism [127]. Consequently, drug–drug interactions are of great concern when having to take other drugs such as antiretroviral treatment while on TB treatment. The most noticeable side effects of rifampicin are that it changes urine, sweat, and tears to an orange-red color. There is no record of adverse effects of rifampicin on the liver when administered in pediatrics [117]. 

Pyrazinamide has sterilizing activity against *Mtb* bacilli by disrupting their energy metabolism and killing the bacteria that persist within the acidic centers of caseating granulomas [123]. Its common side effects include gastrointestinal distress, hepatotoxicity, and joint discomfort; although, the liver toxicity depends on the dose used and treatment duration in adult patients [125]. However, this rarely occurs in children even if it causes minor alterations in their liver enzymes [128]. Ethambutol is bacteriostatic and serves as a complementary drug among this class of medicines. It inhibits cell wall synthesis and offers some protection against drug-resistant mutants [123]. The use of ethambutol is contraindicated in children due to its ability to inflame the optic nerve, thus causing optic neuritis. The diagnosis of this in young children (<5 years old) is difficult since they are unable to comprehend or even articulate their symptoms. Reversal of this adverse effect is possible through treatment discontinuation upon early detection [129].

The following subsections cover second-line (groups 2–4) and third-line (group 5) antitubercular agents utilized for the management of drug-resistant *Mtb* strains. Not all drug models highlighted below are used in pediatric patients as they have been shown to trigger serious adverse events, which are often lethal in children. Normally, complete cure requires lengthy treatment periods; hence, pediatric usage of these bioactives is always with caution bearing in mind their risk-to-benefit ratios. 

##### Group 2 

They are called the second-line anti-TB drugs and are mostly used in the treatment of MDR-TB. They consist of injectable drugs—mainly the aminoglycosides such as amikacin and kanamycin and cyclic polypeptides such as capreomycin and viomycin [130,131,132]. The drugs in this class display bactericidal activity (not sterilizing) and their safety profile is worse [120]. These drugs (amikacin, capreomycin, and kanamycin) are administered over a short course intravenously but can be given intramuscularly when TB therapy is long-term [133]. For the treatment of MDR-TB, aminoglycosides are the drugs of choice with the use of amikacin reserved for last because it has a very potent minimum inhibitory concentration among the aminoglycosides [134]. Capreomycin is reserved for use in the treatment of XDR-TB or for when there is a case of cross-resistance of the drugs belonging to this group. The most adverse effect associated with this group is ototoxicity [112,134].

##### Group 3

They are also classified as second-line antitubercular agents and composed of fluoroquinolone antibiotics, examples of which include floxacin, levofloxacin, moxifloxacin, and gatifloxacin [132]. These antibiotics have good oral bioavailability, are highly active against *Mtb*, and elicit no cross-resistance. Apart from the fact that these molecules can be used for a wider variety of conditions, they have a well-documented history of toxicity ranging from peripheral neuropathy, inflammation and weakening of tendons, dysglycemia, and prolongation of QT interval (which can trigger tachycardia) making them less desirable for use in pediatric patients [112].

##### Group 4

This collection of antitubercular bioactives also falls under the second-line category. Examples of drugs included here are ethionamide, prothionamide, D-cycloserine, terizidone, and para-α-aminosalicylic acid [132]. They exhibit bacteriostatic activity and are thus considered to be less efficacious. Furthermore, their use in children causes adverse effects such as gastrointestinal distress, hepatotoxicity, hypersensitivity, metallic taste, neurological distress, hypothyroidism, psychosis, convulsions, rash, and high sodium load [112,120,135].

##### Group 5

These are regarded as third-line TB antibiotics and are used as remedies for MDR-TB and XDR-TB. They are classified as reinforcing agents, not routinely employed due to concerns about their safety profile after long-term use and limited availability of efficacy data. These drugs should be used in consultation with experts and are contra-indicated in HIV-co-infected patients [53,136]. Examples of drugs in this group include linezolid, amoxicillin/clavulanate, meropenem/clavulanate, clofazimine, and thiacetazone [121,132]. 

#### 4.1.2. Treatment of Non-Resistant TB 

Generally, the treatment of TB in children, without confirmation or suspicion of drug resistance, involves the use of first-line antibiotics, namely, isoniazid, rifampicin, pyrazinamide, and ethambutol [122,137,138]. These medicines are used to treat TB over a period of six months, which comprises the intensive (two months of rifampicin, isoniazid, pyrazinamide, and ethambutol) followed by the continuation phase (four months of rifampicin and isoniazid). The drugs used during the intensive treatment phase are mostly bactericidal in nature and work by rapidly killing ample amounts of the causative bacteria with the overall aim of preventing disease progression, infection transmission, and development of drug resistance. The continuation phase of treatment mainly involves sterilizing agents, which eliminate dormant or intermittently metabolizing bacilli, and often results in complete bacterial clearance, disease cure, and prevention of relapse [139]. Rifampicin and isoniazid function as the principal drugs because they form part of both phases of treatment, while the other two antibiotics (ethambutol and pyrazinamide) are often included based on a variety of patient-related factors [138,139]. 

The treatment regimen for non-resistant *Mtb* strains can comprise of either three or four drugs depending on the location of the TB infection (i.e., pulmonary or extrapulmonary), the patient’s risk of developing resistance to isoniazid, and his/her HIV status [122,137,140]. The “three-drug regimen” encompasses the use of isoniazid, rifampicin, and pyrazinamide for two months in the intensive phase, and isoniazid and rifampicin for four months in the continuation phase. This is often the treatment option for children with suspected or confirmed pulmonary TB who live in areas where the prevalence of HIV is minimal, are HIV-negative, and whose resistance to isoniazid is low [12]. The “four-drug regimen” on the other hand is the regimen of choice for children with pulmonary TB who reside in locations with a high HIV prevalence, are highly susceptible to isoniazid resistance, and have extensive pulmonary disease. This treatment regimen involves the use of isoniazid, rifampicin, pyrazinamide, and ethambutol within the first two months (intensive phase) and then isoniazid and rifampicin in the last four months (i.e., the continuation phase). Ethambutol is included in this regimen because the bacterial load is diagnosed as extremely high and the presence of ethambutol as a companion drug offers increased protection against bacterial resistance [141,142].

Typically, determining the desired dose of TB antibiotics required to provide optimum systemic drug concentrations that would promote therapeutic efficacy without causing toxicity in children and adolescents is usually significantly challenging. This often results in treatment failures and the emergence of drug resistance that ends up being more difficult to manage effectively. The management of the disease at this stage is complicated by the lengthy treatment duration, increased cost (relative to the first-line drugs) and need for injectable drugs that make therapy less tolerable and cause even more severe side effects [143,144]. 

#### 4.1.3. Treatment of Drug-Resistant TB

The selection and/or design of a treatment regimen for children and adolescents is often guided by drug-susceptibility testing (DST) plus the pattern of resistance from the source case [106,145]. In the absence or unreliability of DST, the child and source case treatment history may be used to evaluate the efficacy of the designed treatment plan [28]. Successful cure of drug insensitive TB infection in children is intricate and the use of unfitting therapeutic strategies can have lethal consequences. Management often requires close consultation with experts in the disease who can monitor each therapeutic phase and accompanying adverse reactions or unwanted complications [102]. Generally, the treatment of drug-resistant TB in pediatrics is often guided by the same theories and drug classes as modalities employed in the management of adult patients, even if optimal regimen dose and durations differ on a case-by-case basis [101]. 

As part of the WHO policy recommendations on the design of drug regimens for the treatment of resistant TB strains in children and adults (particularly for RR-TB and MDR-TB), a reordering of TB antibiotics different from the standard classifications was put forward in 2016. Medicines currently used as first-line TB treatment are also contained in this reclassified group because they may play major roles in strengthening the revised course of therapy for drug-resistant infection. This regrouping was based on available clinical evidence, considerations associated with the relative balance between expected and unwanted effects, as well as the practicability of execution. The reordering is often intended as a guide for the longer-term, individualized regimens for patients with drug-resistant TB infection, while shorter-term therapy is standardized putting into consideration the risk–benefit ratio as it relates to the patient [101]. The 2016 WHO guide of revised medicine classifications include: (i) *group A*—the fluoroquinolones, e.g., levofloxacin, moxifloxacin, and gatifloxacin; (ii) *group B*—second-line injectable agents, e.g., amikacin, capreomycin, and kanamycin; (iii) *group C*—other core second-line agents, e.g., ethionamide, prothionamide, cycloserine, and clofazimine; and (iv) *group D*—add-on agents comprising of groups D1 (e.g., pyrazinamide, ethambutol, and high dose isoniazid), D2 (e.g., bedaquiline, delamanid), and D3 (e.g., p-aminosalicylic acid, imipenem/cilastatin, meropenem, and amoxicillin/clavulanate) [146].

Drug treatment for resistant TB strains is similar for pediatric and adult patients. It usually spans across the reclassified groups A–D medicines following a particular fashion ultimately made up of four or even more drugs in some instances. This means that if a regimen is designed such that it includes three drugs from group A, then it should also incorporate one group B drug. If the regimen uses one or two group A drugs, then it should include two drugs from group B. If the regimen cannot be made up of drugs from groups A and B, then drugs from group C or even D can be used [6,101,147]. To choose the most appropriate treatment combination for a patient suffering from RR-TB or MDR-TB, certain selection criteria need to be put into consideration. Some of these key determining factors include [148]:

1. A confirmed susceptibility to or the presumed effectiveness of the medicines that form part of the shorter resistant TB regimen and this requires the exclusion of isoniazid resistance.
2. Patients that have not been exposed to any second-line medicines that are part of the resistant TB treatment regimen for more than a month.
3. Persons that have not acquired tolerance to any of the medicines included in the MDR-TB or RR-TB regimen and have no risk of toxicity or drug–drug interactions.
4. Women that are not pregnant.
5. Individuals that qualify for the RR-TB or MDR-TB treatment regimen if they only have pulmonary TB with no extrapulmonary involvement.
6. The availability of all the medicines involved in the shorter RR-TB or MDR-TB regimen in that specific region or hospital.

Patients who meet the above-listed selection benchmarks are routinely treated with the shortened (nine to twelve months) RR-TB or MDR-TB regimen. while those who do not meet these set criteria will receive the longer term (twelve to eighteen months or more) course of drug therapy, which has been known to increase the probability of cure and lower the risk of chronicity and death. Furthermore, whenever patients placed on the shorter duration regimens, they: (i) experience treatment failure; (ii) do not comply with the prescription; (iii) undergo treatment interruptions for more than two months; (iv) become intolerant to the medicines that are included in the regimen; (v) turn out to be pregnant; or (vi) even experience other conditions that would trigger an exclusion based on the abridged regimen eligibility criteria, it often results in the recommendation of longer duration drug prescriptions. The extended-term treatment plan used in patients not eligible for the reduced duration schedule typically includes a minimum of five drugs with known efficacy against resistant TB strains during the intensive phase. The recommendation usually consists of pyrazinamide and four core second-line TB medicines—one from groups A and B and at least two from group C. If this combination cannot be achieved, groups D2 or D3 bioactive may be added to obtain a total of five agents if not contraindicated in the pediatric age group (for example, bedaquiline is only recommended in individuals over eighteen years; delamanid may also be used in patients aged six to seventeen years) [101,147,148]. 

Whenever any component of the four core second-line medicines and pyrazinamide is considered ineffective, additional groups D2 and D3 agents can be included. This is usually necessitated when resistance to groups A and B is evident (i.e., when a patient has XDR-TB). More drugs can be added to these five depending on their safety profiles and potential to improve the prognosis of therapy. With children suffering from RR-TB and MDR-TB, it is recommended that the prescribed treatment is further strengthened using high-dose isoniazid and/or ethambutol. For those with RR-TB without or unknown isoniazid resistance, it is suggested that the shorter or longer MDR-TB regimen plus isoniazid be utilized [147]. Even though drug-insensitive mycobacterial infection in children is curable, there is still limited data available on the use of most second-line drugs within this age group. This, added to the abovementioned limitations of diagnosis and treatment, means that a shortage of suitable child-friendly dosage forms for second-line TB drugs exists, leading to decreased patient compliance, thus adding to the high morbidity and mortality rate of drug-resistant TB in pediatrics [8,48,103,106,107].

The consolidated drug-resistant TB treatment guideline discussed earlier in this section was updated by the WHO in 2019. This recommendation included a new drug classification, revised parameters for building regimens, improved monitoring strategies, plus a feasible enactment plan based on data generated from clinical trials. Accordingly, through the prioritization of effectiveness and toxicity, a new drug classification that split medicines for drug-resistant TB into three groups (instead of four—A, B, C, and D as detailed above) is detailed below [149,150]:

I. *Group A*: Examples include bedaquiline, linezolid, and the fluoroquinolones, e.g., levofloxacin, moxifloxacin, and gatifloxacin. It is recommended that all three drugs are included as part of the initial therapy unless resistance to these bioactives is evident, or it is not safe to do so
II. *Group B*: Examples are cycloserine or terizidone and clofazimine. They are classified as add-on medicines, and it is recommended that one or both agents from the list is added, except where this is not practical
III. *Group C*: These comprise drugs such as ethambutol, delamanid, pyrazinamide, imipenem/cilastatin or meropenem, amikacin or streptomycin, ethionamide or prothionamide, and para-aminosalicylic acid. They are usually included as part of the regimen when medicines from groups A and B cannot be utilized

#### 4.1.4. Transitioning Landscape in Pediatric TB Therapy

In recent times, there has been a shift toward the use of novel therapeutic strategies based on the regulation of host-immune responses by bioactive molecules. This is geared towards minimizing undue inflammation and immune-facilitated tissue damage that can further complicate TB management in minors. It is becoming clearer that focusing on eradicating *Mtb* would not be the only strategy for improving TB illnesses and deaths in pediatric patients. This is referred to as host-directed therapy and these bioactive molecules can potentially target destructive immunologically directed inflammatory processes (e.g., corticosteroids), augment T lymphocyte-based immunity (e.g., metformin), or modulate immune response by modulating the vitamin D pathway [11]. The use of host-directed therapeutic approaches is a promising treatment solution for pediatric patients but still requires much research as most reported successes are either based on in vitro or in vivo animal studies with very few clinical trials. As such, the use of potential immunomodulators as adjunct TB treatments could promote personalized treatment in children and adolescents [11,151,152].

### 4.2. Diagnosis 

It is a known fact that the diagnosis of tuberculosis in pediatrics is challenging because the symptoms and presentation of the disease mimic those of other viral, bacterial, fungal infections, and/or malnutrition. Furthermore, younger children who are infected with TB often display a myriad of symptoms, both clinically and radiologically, which can easily be mistaken for other common childhood diseases. Depending on a child’s age, the symptoms of active TB are non-specific particularly because they do not appear all at once in most cases, thus making diagnosis in children highly dependent on suspicion of infection by a healthcare worker [56,57,153]. The healthcare practitioner will have to investigate the child’s medical history through inquiries about the child’s household settings as well as the past and current TB statuses of family members and/or caregivers. Following the findings of the healthcare professional, the minor may then be subjected to TB diagnostic testing, which can include chest X-rays, tuberculin skin tests (TST), gastric aspiration, string test, microscopy, nucleic acid amplification tests (NAAT), and interferon-gamma release assays (IGRAs). Other tests such as the GeneXpert *Mycobacterium tuberculosis*/Rifampicin (*Mtb*/RIF) Ultra and GeneXpert *Mtb*/RIF are used if drug-resistant TB is suspected [138,151,154,155,156]. To be able to accurately diagnose TB in pediatrics, biological samples such as sputum, stool, urine, blood, and gastric washings are often required for testing for the *Mtb* pathogen [153]. When pulmonary TB is suspected, biological samples such as sputum are required and are tested using Gene Xpert assays or microscopy; and in the absence of sputum, blood samples are taken for microbiological culture. For extrapulmonary TB, blood samples are required for cell culture assays and urine samples are required for lipoarabinomannan detection [157]. The gold standard diagnostic tool for TB is the culture of *Mtb*. This diagnostic tool is very efficient and accurate in adults but not so in minors because of the paucibacillary nature of the disease in them. Hence, an estimated 70% of all suspected TB cases in children will test negative using this approach [8,158]. 

Furthermore, the paucibacillary nature of TB in pediatrics, history of BCG vaccination, and difficulty associated with biological sample collection because children under the age of seven years lack the tussive force and oral motor coordination that allows sufficient sputum expectoration constitute challenges affecting the accuracy of these test results [138,159]. The collection of sputum is necessary for conducting bacteriological confirmation tests and this is a major challenge because very young children that have pulmonary TB are unable to spit up sufficient saliva for testing purposes. Specimens are thus obtained via gastric aspirate or sputum induction methods, or sometimes, non-respiratory specimens are used depending on the site of disease [160]. Most specimens obtained from children are tested using only smear microscopy, which is not highly sensitive and does not provide information on drug resistance. This is a clear indication for the need to have optimized diagnostic testing for TB, especially in children whose specimens, which are obtained by sputum induction or gastric aspirate, require a highly skilled technician to produce accurate results that can mimic that of spontaneously expectorated sputum from adults [159,160]. A common and most obvious way to optimize specimen acquisition is to obtain multiple samples and perform multiple diagnostic tests on them. However, there are not enough resources, especially in low-income, high-incidence settings, to support this optimization method [9].

In addition, the distinct differences between the pathophysiology and clinical features of the disease in adults and children make proper diagnosis difficult [8,138,161]. There are new diagnostic tests in the pipeline for potentially improving the identification of TB infection in pediatrics. These novel diagnostic assays are designed in such a way that they can detect *Mtb* from biological samples that are easier to obtain from children instead of the traditional sputum, or they rely on the child’s immune response to TB infection. These new diagnostic assays aim to detect TB antigens from host markers or gene signatures under biological or other biorelevant conditions [10].

#### 4.2.1. Recent Advances in Diagnostics for Children

##### Xpert *Mtb/RIF* on Sputum

It is currently one of the most widely used diagnostic tools for pediatric patients. It is an automated cartridge-based PCR assay recommended by the WHO as an initial diagnostic test for presumed pulmonary TB cases in children and adults. The advantage of this diagnostic tool is that it requires minimal sample preparation, can produce results rapidly, and is more sensitive to *Mtb* than smear microscopy when used on sputum and easier to operate than *Mtb* cultures [162,163].

##### Xpert *Mtb/RIF* on Stool

The use of Xpert *Mycobacterium tuberculosis/rifampicin* (*Mtb*/RIF) on stool has proven to have a 32–90% sensitivity and 97–100% specificity to TB antigens when likened to its specificity and sensitivity upon exposure to sputum and/or gastric aspirate samples. The sensitivity of this test is even higher in HIV-positive children as well as in those with severe forms of the disease [164,165,166,167]. Considering the ease of collecting stool samples from pediatrics (particularly those under seven years old) along with these encouraging outcomes, this diagnostic assay stands a good chance of becoming a rule-test in the identification of *Mtb* infection in children. As promising as Xpert MTB/RIF on stool is, it has not been documented by the WHO recommendations for TB diagnosis in children and the procedure for the assay on stool is not standardized [10,152].

##### Xpert *Mtb/RIF* on Nasopharyngeal Aspirates

This investigation employed nasopharyngeal aspirate (NPA) samples, which is desirable because they can be easily obtained with non-invasive techniques. Thereafter, the sensitivity and selectivity of this assay with NPA samples were compared to that of induced sputum (IS) specimens in pairs using the Xpert *Mtb*/RIF and smear liquid culture diagnostic approaches. Summarily, the Xpert analysis showed better sensitivity and specificity for both test samples as well as provided faster results than the culture method. Therefore, testing on two NPA samples can serve as a useful diagnostic tool for testing pediatric patients with suspected pulmonary TB, especially in settings where IS collection and *Mtb* culture are impossible [152,168]. 

##### Lateral Flow Urine Lipoarabinomannan (LF-LAM) Assay 

This is a point-of-care, rapid diagnostic tool that may be useful for pediatric patients. It involves dipping the Determine™ TB LAM Ag test strip into a freshly collected urine sample (60 μL) for the detection of LAM antigen, a lipopolysaccharide present in mycobacterial cell walls. Urine-impregnated test strips are usually incubated at room temperature for about 25 min and changes are inspected by eye following the manufacturer’s guidelines. It is known to be sensitive and specific for detecting TB infection in highly immunosuppressed HIV-positive adult patients and its application in children is an extension of adult data. It has been shown to be 50% and 67% sensitive for TB when compared with culture and Xpert *Mtb*/RIF, respectively, in HIV-positive patients [169,170].

##### C-Tb Skin Test

This novel skin-specific test is based on two immunogenic *Mtb* antigens, namely, the 6-kDa early secretory antigenic target (ESAT-6) and 10-kDa culture filtrate protein (CFP10), which are also used in IGRAs. They are highly specific in BCG-vaccinated healthy individuals [171]. The sensitivity and specificity of this test were found to be equivalent to the tuberculin skin test and IGRAs in patient cohorts that were five years old and above. Its sensitivity was, however, noticed to reduce in HIV-positive patients with a low CD4^+^ count [172,173].

##### T-Cell Activation Marker-Tuberculosis (TAM-TB) Assay

The TAM-TB is a sputum-independent blood diagnostic test that makes use of the characteristic T-cell activation and maturation markers, namely, the CD38 and CD27 to detect active TB infection in children. It is 83% sensitive in pediatrics with culture-confirmed TB and is 96.8% specific for TB in those who were previously classified as not having active TB. The test result is usually available within 24 h [174].

##### RNA Expression Signatures

This assay involved a genome-wide analysis of RNA expression in host blood to determine diagnostic signatures distinguishing active from latent TB and from other diseases. Amongst the participating pediatric cohorts, the assay was sensitive and specific for TB infection in children who either were or were not HIV-positive [175].

##### Gene Signatures

A gene signature can be described as a group of genes expressed differently under specific biological or other conditions such as during active TB infection [175]. Here, a three-gene set (GBP5, DUSP3, and KLF2) in the host whole blood that is robustly diagnostic for active tuberculosis was studied using multiple independent cohorts comprised of children and adults. Overall, the diagnosis was significantly sensitive and selective. Interestingly, the host bacterial drug resistance, BCG vaccination, or HIV status did not interfere with the generated test specificity for active TB and eventual results. With these noted, this assay still requires further evaluation and development before it can be applied in clinical settings [176].

##### Cerebrospinal Fluid Signature Diagnostic Model 

This diagnostic assay uses VEGF, IL-13, and cathelicidin LL-37 to detect TB meningitis in children. It has been proven to be 52% sensitive and 95% specific for TB meningitis. The findings from studies involving CSF signature suggest that it is a promising diagnostic tool for TB meningitis and could help guide strategies for preventing the immunopathology that is associated with TB meningitis [177].

##### Truenat Assays (Mtb, Mtb Plus, and Mtb-Rif Dx)

These new molecular diagnostic tools were developed by Molbio Diagnostics in India and they are used for the detection of active TB infection as well as rifampicin resistance. The sputum serves as the test sample in this case. The test utilizes a chip-based real-time micro-polymerase chain reaction (PCR) for the identification of TB and rifampicin resistance from deoxyribonucleic acid (DNA) that is isolated from the sputum sample collected [178]. The WHO included it in a recent guideline publication as an acceptable initial diagnostic test for TB in adolescents fifteen years and above rather than using the culture/smear microscopy approaches [152]. 

##### Type 1 Cytokine Plasma Signatures as Biomarkers

Kumar and colleagues conducted a prospective diagnostic study to identify biomarkers for the diagnosis of TB in pediatric patients. In the study, a total of one hundred sixty-seven children, aged between five and ten years old, were recruited and classified under three cohort groups: confirmed TB, unconfirmed TB, and children with other respiratory ailments with tuberculin skin positive or negative as unlikely TB controls [179]. In this study, multiplex ELISA was used to examine the plasma levels of cytokine type 1 biomarkers (TNFα, IFNγ, and IL-2) and type 17 (IL-17A) and other pro-inflammatory (IL-6, GM-CSF IL-1a, and IL-1b) cytokines as biomarkers in all cohorts. They then made the discovery that the plasma levels of these cytokine biomarkers were higher in active TB infection (confirmed and unconfirmed cohorts) as compared to those in the unlikely TB controls. Upon further analysis of the behavior of these cytokines using the Receiver Operating Characteristics (ROC), they found that IFNγ, IL-2, and IL-17A in the unconfirmed TB cohort and TNFα and IL-17A in the confirmed TB cohorts could possibly serve as biomarkers to differentiate active from unlikely TB cases [179]. The biomarkers displayed a 100% sensitivity and a specificity of between 98 and 100%, which meets the specified target profiles set by the WHO [180]. This discovery is suggestive of the possible use of these biomarkers in the development of a rapid point-of-care test for the diagnosis of TB in the pediatric population [179]. 

Despite all that is currently available, in use and under investigation, to detect TB, there is still the need to improve the prediction of TB susceptibility, diagnosis, and treatment outcomes of the disease, especially in children. To meet this need, the diagnosis, prediction of susceptibility, and treatment outcomes in children need to be managed using an individual approach instead of a “one-size-fits-all”. It is important to bear in mind that satisfying this need requires the use of biomarkers and immunological signatures, which all depend on factors such as genetic, age, nutrition status, drug-resistant *Mtb* strains, co-infections, BCG vaccination status, etc., all of which affect an individual’s gene expression [153,181]. The use of biomarkers plays an important role in clinical decision making to monitor TB treatment in individuals. This is a very promising approach; however, it is inauspicious in real life because it is expensive, extensive, requires a higher level of expertise, and therefore may not be applicable in the resource-poor settings in which TB prevails [182].

### 4.3. Prevention 

Commonly used strategies for preventing TB in the cases of pre-exposure or post-exposure, latent TB infection, and secondary prophylaxis (which is only given after the patient has completed TB treatment) include drug therapy and vaccines [123]. Inactive TB infection predisposes pediatrics, particularly those under five years old, to a higher risk of developing the active disease in their lifetime compared to adults [108,183]. Tuberculosis preventive therapy will thus be most beneficial to this group of younger persons and, as such, should be targeted and tailored for them [6]. Children who have been exposed to TB but have not been diagnosed with active TB have various prophylactic treatment options that are available to them. The challenge here is that most of these treatment options have not been tested against each other, thus making it difficult to select the optimum treatment regimen. This leaves the selection of regimen to be based on affordability, availability, adverse events, health professional choice, and patient compliance. These approaches for TB preventive therapy are guided by specific recommendations set by the WHO for different categories of pediatric patients based on their risk of contracting active TB infection.

#### 4.3.1. General Prevention Guidelines for Pediatrics

##### Adolescents Living with HIV

Adolescents who are HIV-positive receive antiretroviral therapy because this remains the standard for management irrespective of their TB status. Despite this, TB still accounts for a third of all HIV-related deaths, adolescents inclusive [6]. The Guideline Development Group (GDG) recommends TB preventive therapy for all adolescents living with HIV irrespective of the strength of their immune system or whether they are symptomatic, asymptomatic, or receiving ARTs. Abnormal radiographs on chest X-rays constitute another factor that warrants the initiation of TB preventive therapy in this group of individuals [131,184]. The GDG also recommends immediate TB prophylaxis for people living with HIV who have successfully recovered from TB. This recommendation, however, does not include those who have successfully recovered from MDR-TB and XDR-TB since studies in this regard are still ongoing [184]. 

##### Human Immunodeficiency Virus-Positive Pregnant Women and Their Fetuses 

Both pregnancy and HIV increase the risk of contracting TB, which can result in poor and unwanted birth outcomes. Pregnant women who are HIV-positive have a higher risk of TB infection and they may pass the disease onto their fetus. This can result in the death of both mother and fetus [109]. It is worth noting that the key drugs in TB preventive therapy, namely, rifampicin and isoniazid are classified as category C in pregnancy, meaning that animal reproductive studies have shown the occurrence of adverse impact on the fetus, and that well-controlled studies have not been executed in humans. Thus, the potential benefits associated with their usage necessitate their continuous use in pregnant women, despite the possibility of developing fetal adverse effects [106]. Recent studies executed by Gupta et al. [185] found that even an isoniazid-only prophylaxis begun during pregnancy or 12 weeks post-delivery led to higher risk of poor health outcomes as well as fetal and newborn mortalities. 

##### Infants and Children

According to recent WHO recommendations, infants that are household contacts of TB infected persons, younger than twelve months old, and who upon appropriate clinical evaluation show no sign of possible active TB should receive preventive therapy. Generally, since infants and children display symptoms different from adolescents and adults, a history of contact with a TB-infected individual(s) would require that the minor be tested for TB infection and diseases that produce similar symptoms. Even if active TB infection is ruled out after testing, preventive treatment remains highly recommended. HIV-infected children older than twelve months or who reside in high TB transmission zones require prophylaxis treatment even if symptoms of active disease are absent or if they had successfully been treated previously. Lastly, HIV-negative children who have household contacts for bacteriologically active pulmonary TB also require preventive treatment even if no signs of active pulmonary TB are presented upon clinical examination.

#### 4.3.2. Chemoprophylaxis in Pediatrics

Tuberculosis preventive therapy or chemoprophylaxis is often administered to children under the age of five or children of any age who are HIV-infected after exposure to TB infection or if they are re-exposed to TB through close contact with an infected person. It provides excellent protection against the development of tuberculosis and reduces the risk of children (less than fifteen years of age) developing TB by up to 59% in the absence of drug resistance [34,56,186,187,188]. There are several recommended options that can be explored for preventing active TB infection in the pediatric population and, some commonly recommended regimens are summarized as follows:

1. Six or nine months of daily isoniazid [189,190], which has been shown to reduce the risk of TB infection by up to 36% [191]. The nine-month isoniazid monotherapy is known to be more efficacious than the six-month preventive therapy; hence, it is the preferred alternative [152].
2. Weekly rifapentine plus isoniazid taken for three months in total produces pharmacological effects that are equivalent to six or nine-month isoniazid monotherapy. This is often recommended for children two years and older, including HIV-positive persons. Limited pharmacological data on the use of rifapentine in children younger than two years old. This is the preferred TB preventive regimen in countries with a TB incidence of <100 per 100,000 population [2,27,152,192,193].
3. A regimen of four-month daily rifampicin is a preferred alternative prophylaxis strongly recommended for HIV-negative children of all ages; although, there is no evidence of efficacy in HIV-positive groups. This regimen is known to have similar clinical efficacy to the standard six or nine-month daily isoniazid monotherapy. It is safer than the isoniazid monotherapy because its risk of causing hepatotoxicity is lower [2,192,193].
4. Three-month daily rifampicin plus isoniazid is recommended for infants and children under fifteen years because the benefits of use within this age group outweigh the potential risks. Moreover, child-friendly fixed-dose combinations of rifampicin and isoniazid are commercially available, and this generally favors patient compliance. This combination can also be used in HIV-positive as drug–drug interaction with antiretroviral agents (e.g., lopinavir/ritonavir, dolutegravir, and nevirapine) permit [28,193,194].
5. Adolescents with unknown or positive tuberculin skin test results, who are HIV-positive, considered unlikely to have active disease despite exposure, and resident in resource-constrained TB endemic/high TB transmission areas, should receive at least the thirty-six-month daily isoniazid preventive therapy. The isoniazid preventive therapy should be administered to such individuals regardless of whether they are on ongoing antiretroviral therapy schedules or not, their level of immunity, previous TB treatment history, and their pregnancy status [21,195].

#### 4.3.3. Vaccine

Bacille Calmette–Guerin (BCG) remains the only available vaccine that is used for TB prevention in the world. It is particularly the primary mode of preventing TB in children [2,34,186,196]. It contains a live, attenuated strain of *M. bovis*, which is the primary cause of TB in cattle. Protection against severe forms of TB, especially tuberculous meningitis and disseminated TB in infants and young children can be provided by BCG vaccination [197]. It does not prevent primary infection and reactivation of latent pulmonary infection, the main source of *Mtb* community spread, thus limiting the impact of BCG vaccination on transmission. Globally, BCG is known to be ~50% efficient in preventing severe disseminated diseases, such as TB meningitis and miliary TB, and is 0–80% effective in providing protection against pulmonary TB [8,198,199]. The efficacy of the BCG vaccine is influenced by the immunogenicity of a specific strain, administration technique used, patient age at the time of vaccination, population genetic differences, host’s day-to-day nutrition, co-infection with parasites, exposure to environmental mycobacteria, and genetic variations in *Mtb* strains [8,200,201]. 

Considering that BCG is a live vaccine, the absence of an efficient immune response, such as in immunocompromised, HIV-positive children, presents a 1% chance of developing into disseminated BCG disease, which has an all-cause mortality rate of 75–86% [34,197]. Consequently, the WHO advises against administering the BCG vaccine in HIV-infected children [202]. Reports from clinical studies have shown that childhood BCG vaccination that triggered scar formation decreased the risk of adulthood TB by 70%, regardless of the patient’s HIV status. In instances when disseminated BCG disease occurs, appropriate TB antibiotic combinations should be administered considering the inherent and variable resistance to pyrazinamide and isoniazid, respectively. Discontinuation of the BCG vaccination had a negative impact in low-incidence countries such as Sweden. The universally adopted strategy of replacing BCG inoculation with directed vaccination programs significantly reduced coverage, which led to a 15-fold upsurge in TB incidence in children from foreign parents [34,203]. 

The oldest and only used vaccine for TB in children is the BCG vaccine [34,196]. As successful as the BCG vaccine has been over the years, it does have its own challenges and it could benefit from some modifications or optimization. It would be a great benefit to children if they were to have a vaccine that is active against severe childhood TB and can protect against adult forms of TB because this would limit TB transmission significantly. With this need having been noticed, stakeholders have devised novel vaccine strategies such as developing—(i) mycobacterial whole cell-derived vaccines derived from *Mtb*, BCG, or closely related strains of non-tuberculous mycobacteria, which are often classified as live vaccines that have been attenuated through genetic modification or vaccines derived from killed or fractionated whole mycobacteria; (ii) subunit vaccines which can be subdivided into adjuvanted protein subunit vaccines and recombinant viral-vectored vaccines; (iii) adjuvanted protein subunit vaccines that contain an antigenic protein, or a linked series of antigenic proteins formulated along with immune-stimulating adjuvants; and (iv) recombinant viral-vectored vaccines, which are designed to activate a robust and long-term immune response to the antigens presented, excluding the need for an exogenous adjuvant [204,205].

### 4.4. Miscellaneous Issues

Over the years, the importance of TB control has become even more evident. As such, the WHO, government, various other organizations, and world leaders have embarked on a mission to come up with ways to curtail its spread globally. One of such strategies is the Directly Observed Treatment Short-term (DOTS) strategy, which was developed as part of the WHO’s END-TB strategy. This strategy was aimed at promoting high TB cure rates by providing inexpensive diagnostic tests for smear-positive TB patients, as it is difficult to predict the development patterns of the disease because of limited resources and prolonged latent infections that make diagnosis challenging. The DOTS approach encompasses four main aspects including: (i) detection of smear-positive pulmonary TB in patients from public clinics with limited resources through sputum smear microscopy in a quality assured laboratory; (ii) provision of directly observed treatment with short-course chemotherapy; (iii) guaranteeing a continuous supply of anti-TB drugs; and (iv) using case study recording to keep track of treatment outcomes [206]. The findings from the DOTS strategy have made it easier to predict TB transmission patterns, eased the detection of TB as well as increased TB cure rates in large populations, thus ensuring effective control of TB [207,208].

Despite the success of the DOTS scheme, there are still cases whereby pediatric patients are lost through migration. In this case, treatment success is mainly dependent on the parents’ compliance. This led to the development of the Integrated Maternal Mobile Health (IMMH) by Kenyan scholars. This approach employed Global Positioning System (GPS) to track pregnant women at risk of transmitting TB to their newborns to ensure that they receive proper antenatal care, laboratory, and vaccination services to avoid missing scheduled treatment. A case study showed how implementation of the IMMH method produced successful treatment outcomes for a child diagnosed with TB at nine months. The IMMH incorporation with the DOTS enabled the child to achieve full recovery in four months [209]. The use of such innovative systems is necessary to bridge the gaps between case detection, treatment, and prevention of TB in children. However, without intensified political will, the lack of resources will affect the successful implementation of such systems in high-burden regions [10].

## 5. Pediatric Tuberculosis and Pharmaceutical Dosage Forms

Pediatric dosage forms refer to pharmaceutical formulations or drug products that are appropriate for safe, accurate, effective, and adherent administration of medicines to children of various ages [210]. The treatment outcome for TB disease in children is more promising than it is for adults. 

However, about 205,000 deaths in HIV-negative and approximately 32,000 deaths in HIV-infected children and adolescents are linked to TB disease [23,27,28,211]. This was partly because of the significant shortage of suitable dosage forms for TB treatment in pediatric patients, which commonly results in non-compliance and treatment discontinuation, which ultimately leads to therapy failures and the development of drug resistance [23,27,28,212]. 

With TB drug treatment, children are treated as miniature adults instead of the uniquely diverse population that they are. Largely, the development of most commercially available dosage forms is executed with adult patients in mind. This makes them unsuitable for the much-needed flexible dosing or difficult to adapt to dose modification based on age and/or weight, which is routine for pediatric TB management. This neglects the fact that children differ from adults in terms of their biology, physiology, and pharmacokinetics; thus, what is suitable for an adult may be inappropriate for pediatrics and vice versa. Recent studies have found that current clinical practices involving dose extrapolation from adult drug products often generate sub-therapeutic plasma levels in children, particularly when compared with the dosing guidelines recommended by the WHO for pediatric TB management. Despite these concerns, pharmaceutical companies are yet to produce more child-friendly dosage forms for the treatment of TB in children. This is often influenced by the fact that the market for pediatric dosage forms is relatively small, risky, and less profitable than that of adults [213,214]. 

With the known global shortage of pediatric TB medicines, off-label use of TB medicines is common practice. This usually involves the crushing or splitting or breaking of adult solid dosage forms into sizeable portions or even powders for administration with water, milk, juice, or even soft food to accommodate the pediatric patients. In some instances, physicians prescribe extemporaneously compounded antitubercular formulations for children, and the common challenge often associated with these kinds of orders is that their preparation and concentrations tend to vary from pharmacy to pharmacy and even one hospital to the other ([7,215,216,217]). These administration methods can potentially result in dosing inaccuracies, unwanted drug–food interactions, reduced drug potency, impaired formulation stability, disruption of formulation coating/external layering, compromised bioavailability, and inconvenience associated with caregiving, which ultimately leads to poor patient compliance, undesired pharmacotherapeutic outcomes and eventual onset of resistance [7,112,218,219,220]. 

Of the first-line TB antibiotics used in children, only isoniazid and rifampicin are commercially available as single-entity pediatric formulations. Other dosage forms that have been explored include double-fixed dose combinations (FDCs) of isoniazid and rifampicin, triple FDCs of isoniazid, rifampicin, and pyrazinamide, as well as FDCs containing all four first-line drugs, i.e., isoniazid, pyrazinamide, rifampicin, and ethambutol. Other quality-assured pharmaceutical dosage forms for the treatment of TB have not yet been successfully explored up to the commercialization phase, particularly the second-line drugs, which are now required by the growing number of children infected with the drug-resistant TB strain worldwide [216,218,221]. Scientists are continuously working toward improving pediatric antitubercular formulations, particularly the fixed-dose combinations, by optimizing the dosing parameters in a way that makes the medications effective and suitable for use in children of all age groups [118,218,222]. 

### 5.1. General Considerations for Pediatric TB Drug Formulation

Pharmacotherapy in pediatric patients is remarkably diverse because, throughout childhood, they grow continuously, their bodies change rapidly, organs mature, and they develop better cognitive functions [223]. The design of age-appropriate pharmaceutical formulations for children is challenging because of the differences in the developmental stages that they go through [223]. Therefore, formulating novel dosage forms that suit the needs of pediatrics in terms of their age, size, physiologic condition, and specific treatment requirements is vital [224,225]. According to the International Conference on Harmonization (ICH) guidelines, pediatrics can be classified as preterm newborn infants, term newborn infants (zero to twenty-seven days), infants and toddlers (twenty-eight days to twenty-three months), children (two to eleven years), and adolescents (twelve to sixteen to eighteen years based on the region). The Food and Drug Administration, on the other hand, has categorized pediatrics as neonates (birth to one month), children (two to twelve years), and adolescents (twelve to less than sixteen years) [226,227]. All these groups differ in terms of their cognitive abilities and capacity to use different dosage forms. Each group displays uniqueness in their pharmacodynamics and pharmacokinetic profiles, gastric acidity, intestinal motility and conjugation, and transport of the bile salts as compared to adults. It is thus important that drugs developed for the treatment of pediatric TB are designed to suit the different target pediatric subpopulations in terms of safety, efficacy, and drug quality [226,228]. 

The route of administration and nature of the pharmaceutical formulation are particularly important factors for determining pediatric patients’ compliance with prescribed medications. If the administration of a drug formulation is cumbersome, causes pain or discomfort to the child, or requires the consumption of multiple doses, it is highly likely that the caregiver would not comply with the prescription [222,224]. For older children and adolescents to conform to medication regimens, the dosage form and route of drug administration should not compromise the child’s lifestyle so much that peer influence can discourage them from adhering [222]. The dosage form of choice should address the problems of palatability, ease of swallowing, safety, good quality, and affordability. Other vital factors that can be considered for optimal pediatric TB drug formulation include: (i) children’s vulnerability during clinical trials; (ii) challenges associated with effective taste-masking, particularly for oral dosage forms; (iii) limitations related to the types of excipients that can be safely used; and (iv) the lack of suitable manufacturing technologies specific for making such specialized formulations [227,229]. 

The oral route of drug administration remains the most common and preferred for the management of TB infection in pediatric patients. Most prescribed TB medicines are in the form of oral solid dosage forms such as single-entity and fixed-dose combination tablets and liquid preparation in the form of solutions, suspensions, etc. [215,230]. The solid dosage forms are mostly inappropriate for pediatric dosing and usage. They often have limited dose flexibility and unpleasant tastes despite taste masking, cannot be swallowed by younger children (particularly those under five-year-old), may pose choking hazards, and often result in caregivers having to crush them. It has also been reported that even older children, up till the age of eighteen, still struggle to swallow tablets that are more than 15 mm in diameter. These points further promote the “off-label” use of TB medicines and exacerbate the effects of inaccurate dosing that can eventually render the medicine ineffective, and potentially trigger drug resistance [7,227,231,232]. Liquid antitubercular preparations, on the other hand, are more child friendly and easier to swallow, but are often limited by their poor palatability and lack of dose uniformity, often resulting in the need to use more excipients such as sweeteners, stabilizers, etc. These excipients can improve their chemical, physical, and microbiological stability, but may not be suitable for pediatric application [7,218,222,224]. To successfully treat TB in pediatric patients, it is important that specially designed, age-appropriate pharmaceutical formulations are available to ensure safe and efficient pharmacotherapy [233,234,235]. Furthermore, the inclusion of children in clinical trials would also go a long way in strengthening the development of these specialized formulations as rigorous pharmacokinetics, pharmacodynamics, optimal dose/dose-finding, efficacy, and safety profiling evaluations can be executed [236,237].

### 5.2. Advancements in Pediatric Antitubercular Drug Formulation 

It has been reported that less than fifty percent of all the drugs that are currently being prescribed for children infected with TB are not commercially available in forms suitable for pediatric consumption [7,218,230,238]. This can be attributed to the fact that the market for pediatric dosage forms is relatively small, risky, and less profitable than that for adult pharmaceutical formulations [213,227]. Further, the development of age-appropriate, anti-TB pharmaceutical formulations for children is challenging because of the differences in their developmental stages and effective taste masking problems, which greatly impacts compliance as well as limitations surrounding safe excipient selection and large-scale manufacturing [223,226,229]. The most commonly available branded dosage forms for TB treatment in pediatrics include oral solutions, suspensions, and single and fixed-dose combination solid dosage forms [215,218,230,239]. In most cases, adequate drug management of TB infection in children can be cumbersome because the treatment is entirely comprised of antimicrobial agents with unpredictable permeability, solubility, absorption and stability, unpleasant taste, plus almost inevitable side effects, especially when developed into different pharmaceutical formulations for patient consumption. These possible glitches are mostly attributed to the differences in the selected administration routes, systemic drug concentration, therapeutic index, and treatment duration, which significantly influences drug performance and pharmacological efficacy [240,241,242]. 

Scientists are therefore constantly exploring new delivery strategies capable of improving currently available antitubercular drug formulations, to make them less burdensome for pediatric patients’ day-to-day use, as well as to increase drug concentrations at affected sites, optimize therapeutic index, and reduce toxic effects and treatment duration to achieve the desired treatment outcomes [7,218,227,242]. Different novel drug delivery approaches such as electrospun orodispersible film [219], co-polymeric orodispersible formulation [218], fixed-dose combination orally disintegrating tablets [243]; reconstitutable dry suspension [7]; water-dispersible solid preparations [216,241]; liquid fixed-dose combination polymeric micelles [244]; multi-component extemporaneous powder for suspension [245]; 3-D printed tablets for personalized dosing, which allows dose adjustment according to the patient’s weight and metabolic rate [246]; cyclodextrin-based oral solutions [247], hot melt extrudates [248], etc., have been explored for pediatric delivery of antitubercular agents. Even though most of these advancements are still in the research and development stages, these all signify meaningful contributions to the global management of pediatric TB.

## 6. Discovery of Novel Compounds for Tuberculosis Therapy in Pediatrics

There is a rise in the incidence of drug-resistant TB, and this is evidenced by the increase in multi- and extensively drug-resistant strains in both adults and children. As previously stated, this is the resistance of the strains to one or two of the first-line and at least another second-line anti-TB drug. This makes it clear that the existence of the five classes of drugs, which include first-line treatment of drug-susceptible TB, as well as drugs that can be used in combination to treat drug-resistant infection, is not enough to provide a total cure [104,249]. The treatment of TB relies solely on antimicrobial agents, which are among the most delicate of all drug categories because their use increases the probability of treatment-induced drug resistance [250]. Consequently, researchers and clinicians are constantly seeking innovative methods of synthesizing novel antitubercular agents that can find use as therapeutic and/or prophylactic drugs, particularly for pediatric patients. The repurposing of existing bioactive molecules for use as antitubercular agents and the identification of chemotypes that are being evaluated for their efficacy in in vivo studies are also being explored for the same purpose [251,252,253]. 

### 6.1. New Drug Molecules 

Bedaquiline and delamanid are new drugs that have shown acceptable outcomes in the treatment of drug-resistant TB strains in children and adolescents, but unfortunately, there is limited access to these remedies [254,255,256]. 

#### 6.1.1. Bedaquiline

Bedaquiline is the first antituberculosis drug approved by the Food and Drug Administration (FDA) since the late 1970s. It is classified as a diarylquinoline and registered by Janssen Pharmaceuticals as Sirturo^®^. It is a strong bactericidal agent that acts by interfering with the mycobacterial ATP synthase and is known to be effective against drug-resistant *Mtb* strains when included in TB regimens [256,257,258]. It is selective for *Mtb* in that its c-subunit differs from that of humans by three amino acid residues, which creates a binding site that is unique to bedaquiline [259,260]. It thus acts on this c-subunit as an inhibitor of ATP synthase within the mycobacterium, resulting in a depletion of ATP and a pH imbalance within the *Mtb* structure. It has been reported that bedaquiline exhibits its effect potently and selectively for both drug-sensitive and drug-resistant strains of *Mtb,* having a minimum inhibitory concentration (MIC) of 0.03 mg/mL for drug-sensitive and 0.12 mg/mL for drug-resistant strains for both chronic and acute TB [261]. 

Pharmacokinetic studies in humans, particularly adults, have revealed that post single oral administration, bedaquiline is well absorbed with an average peak plasma concentration time of 5–6 h and an effective half-life of greater than 24 h. Its lengthy terminal half-life in human beings makes alternating drug administration schedules possible when used in combination with other TB antibiotics as part of the MDR-TB and XDR-TB regimens [256]. The initial clinical trials on the use of bedaquiline for the treatment of drug-resistant TB for its approval by the WHO in 2013 excluded children under the age of eighteen [262]. However, due to its effectiveness, the interest it has generated, and the need for the inclusion of children in clinical studies, several trials assessing its safety and pharmacokinetic profile in pediatrics have been executed [105,144,256,263].

Bedaquiline is currently available in 100 mg tablets and 20 mg dispersible tablets [256,263]. With the provisional study outcomes generated to date, the WHO, and FDA approved the use of bedaquiline as a substitute for second-line injectable drugs in children above five years old if they weigh more than 15 kg. The recommended dose for bedaquiline in children fifteen years and under that weigh 16–30 kg is 200 mg daily for 14 days, and then 100 mg daily on Monday, Wednesday, and Friday for 22 weeks while those weighing greater than 30 kg will receive 400 mg daily for two weeks followed by 200 mg three times weekly over 22 weeks [256].

#### 6.1.2. Delamanid

Delamanid is an antimicrobial agent from the class of nitroimidazole. It exerts its effects on non-replicating *Mtb* by using deazflavin-dependent nitroreductase (F420 coenzyme system) to release nitric oxide within the cell, which then disrupts the biosynthesis of mycolic acid (i.e., methoxy-mycolic acid and keto-mycolic acid) resulting in the depletion of mycobacterial cell wall apparatuses and eventual bacterial death [256,264]. It has been shown to have early bactericidal activity in humans and in vivo studies using human models/animals have shown that delamanid can clear the lungs of bacteria faster than the current treatment regimen for drug-resistant TB [256,260]. Delamanid absorption was observed to be higher, almost twofold, when administered with meals compared to the fasted state. Its pharmacokinetics were described as nonlinear, meaning that a doubled dose results in less than twofold efficacy. Peak plasma concentration is around 4–8 h post-oral administration with a half-life of 30–38 h. It has a large volume of distribution with a total protein binding of ≥99.5%. Its metabolism is primarily by albumin (i.e., a non-hepatic route) and excretion is mainly through stool with approximately 6% excretion in urine. In vivo studies on animal models showed that delamanid molecules can permeate brain and placental blood barriers as well as be excreted in breast milk [256,265]. 

Most clinical trials on delamanid have been conducted on adults while data on its safe use in children are still generally underway as many studies are recruiting participants or are ongoing. The few case studies captured in the literature did not report a high incidence of adverse effects; however, the limited availability of robust clinical data makes it difficult to draw firm conclusions on its pharmacokinetics and safety in pediatric patients. It is therefore important to highlight that careful monitoring and further investigations in pediatric cohorts are essential for establishing its therapeutic efficacy, good tolerability, and the development of optimal dosing parameters [256,266]. An interim policy guidance was provided by the WHO on the usage of delamanid in the management of TB in children and adolescents infected with the drug-resistant *Mtb* strain. This policy recommended the inclusion of delamanid in the primary MDR-/XDR-TB regimen for pediatric patients who were previously treated with second-line TB antibiotics; or those having typical isolates with additional resistance to fluoroquinolones or second-line injectable agents; or instances in which the use of the shorter MDR-TB regimen was contraindicated. Considering the scarcity of sufficient data on the use and safety of delamanid plus the need for further research and more evidence, current WHO guidelines classify it as a Group C drug in children three years and older [147,256].

It was reported that, in practice, the recommended dose for delamanid is 25 mg twice daily in children aged three to five years old who weigh <24 kg; 50 mg twice a day for children six to eleven years old who weigh 24–34 kg, and in older children aged twelve to seventeen years old weighing >35 kg, 100 mg two times a day after meals. Delamanid is available as a 50 mg tablet and does not come in any other pediatric-friendly dosage form. Dose adjustments for its administration to pediatrics involve crushing and mixing the tablet, which compromises its stability and bioavailability, thus necessitating its administration by an expert. Moreover, its contents are bitter leading to reduced acceptability and patient compliance considering that palatability of medicines is crucial for pediatric medicines [255,256].

### 6.2. Repurposed Drugs

The repurposing of existing antibiotics for the treatment of TB in children involves optimizing existing drugs that are known or have been proven to be safe for use in children and are well tolerated by this population of individuals. An estimated 33,000 children develop MDR-TB on a yearly basis [255,267,268]. Even though children have a greater potential to recover from MDR-TB, they are also more at risk of suffering the severe adverse effects associated with the existing second-line MDR-TB drugs [25,256,269]. New drugs such as bedaquiline and delamanid, as well as the repurposed drugs such as linezolid and clofazimine, have been proven to be effective in the treatment of MDR-TB in adults, but have not been studied as extensively in pediatrics, and as a result, they are not very accessible to them [270,271]. The lack of pharmacological data, because of the exclusion of children and adolescents in clinical trials, means that healthcare workers catering for this patient category lack the appropriate dosing and safety information that is necessary for pharmacotherapeutic management [267]. This leaves them with the challenge of having to administer these new and repurposed drugs to children at their own discretion [255]. More studies on the use of this class of bioactives are beneficial when clinicians need to make decisions on their use in pediatric patients [258].

#### 6.2.1. Linezolid 

Linezolid is an oxazolidinone antibiotic that is known for its efficacy against Gram-positive bacterial infections. It has also been shown to be effective in the treatment of MDR-TB in adults. Clinical data generated for pediatric patients, though limited, have shown promise of good efficacy despite extensive disease, significant drug resistance, and failed therapy with the use of second-line TB antibiotics. Notably, linezolid is highly lipophilic and permeates the cerebrospinal fluid (CSF) well, and has been shown to facilitate CSF sterilization, making it an important component of MDR-TB meningitis regimens. It has been recommended that it should be included as a core drug (of at least four agents) in the management of MDR-TB in children, particularly in instances where fluoroquinolone resistance exist or other second-line injectable drugs are contraindicated. Its use is also repurposed for the treatment of XDR-TB in children [258,270,271,272]. Linezolid has good bioavailability, approaching 100%, after oral administration of both its 20 mg/mL suspension and 600 mg tablet formulations. According to the literature, a dose of 10 mg/kg given to children ages three months to less than twelve years old yields peak plasma concentrations that are relatively close to what is recorded post administration of the adult dose of 600 mg. Generally, the recommended dose is 10 mg/kg once a day in children twelve years and older; and 10 mg/kg twice a day in children younger than the age of twelve indicating that careful dosing is essential for this age group to minimize adverse reactions [255,258]. 

The use of linezolid should be restricted for short durations because of its toxicity profile particularly myelotoxicity, gastrointestinal complications, and peripheral and optic neuropathy when used over a prolonged period [258,273,274]. Linezolid toxicity is dose dependent, and research has shown that children tend to experience fewer adverse events from its use compared to adults. Peripheral neuropathy can be irreversible and may be quite challenging to identify in pediatric patients. Common gastrointestinal disturbances in this age group include diarrhea and vomiting, and these rarely need dose cessation [258].

#### 6.2.2. Clofazimine

Clofazimine is a highly lipophilic riminophenazine antibiotic commonly used for treatment of leprosy and has been repurposed for the treatment of MDR-TB in children. Pharmacological data for its use in children for the treatment of leprosy (caused by *Mycobacterium leprae)* are well known, and it has been proven to be well tolerated by children [240,258,275]. For the treatment of pediatric MDR-TB, clofazimine is given in 2–3 mg/kg daily doses and every other day in younger children who require smaller doses (up to a maximum dose of 100 mg daily). This staggered, yet effective dosing in younger children is feasible because of its long half-life. Clofazimine is commercially available as 50 and 100 mg gelcaps, which are pharmaceutical preparations that cannot be crushed or split making dosing challenging for pediatrics. In humans, clofazimine extensively binds to plasma proteins and has an extremely lengthy half-life that can span from days to months depending on the administration duration and dose with a tendency of triggering tissue accumulation and crystallization that can lead to toxicity such as stigmatizing skin discoloration [255,258,276].

#### 6.2.3. Miscellaneous

Fluoroquinolones such as ofloxacin, levofloxacin, moxifloxacin, and gatifloxacin form part of the drugs that have been repurposed for the treatment of TB in children. Ofloxacin and levofloxacin are often reserved for use as second-line treatment in MDR-TB, whereas moxifloxacin and gatifloxacin have been considered as substitutes for ethambutol or isoniazid for the treatment of drug-susceptible TB [277]. The literature has also reported the use of fluoroquinolones as an effective prophylaxis for MDR-TB in both adults and children under twelve years old, particularly with high-risk contact/exposure [258]. Reported adverse effects associated with the use of fluoroquinolones for the treatment of drug-resistant TB are mostly in adults with a negligible incidence in children. Generally, fluoroquinolones are considered safe for use in children, even those less than five years of age, who are taking them longer term (six months therapy). In addition, as a key bactericidal drug component of drug insensitive TB regimens, with treatment terms longer than twelve months in some instances, fluoroquinolones are still considered well tolerated in pediatric patients. However, only the use of levofloxacin resulting in self-limiting musculoskeletal ailments was documented [150,258].

Rifapentine is another drug repurposed for the treatment of TB in pediatrics; it is a rifamycin antibiotic and a potent inhibitor of mycobacterial activity. It has been shown to be an effective antibiotic against various other bacterial infections. The replacement of the core drug rifampicin with rifapentine is an attractive alternative to reduce side effects and to shorten the duration of TB treatment [278]. Anti-TB regimens comprising of rifapentine tested in adults and adolescents over the age of twelve (HIV-positive and HIV-negative) have demonstrated a two-month reduction in the duration of anti-TB treatment [279]. There is, however, a lack of data regarding the safety, pharmacokinetics, and dosing of rifapentine, and the presence of a nitrosamine impurity in rifapentine hinders the success of studies that are to investigate these factors. 

## 7. Impact of Coronavirus Disease on Tuberculosis in Children 

Severe acute respiratory syndrome coronavirus-2 (SARS-CoV-2), otherwise known as the coronavirus disease 2019 (COVID-19), is a respiratory illness with signs ranging from that of a common cold to more severe forms of disease with symptoms mimicking those of pneumonia [280,281,282]. This virus spreads through droplets that are released when an infected person coughs or sneezes and is transmitted to another person by means of inhalation and/or touching infected surfaces [283,284]. The symptoms of COVID-19 infection are noticeable within 3–5 days post-infection [285] and even those infected individuals who are pre-symptomatic or asymptomatic can transmit COVID-19 infection. The infection transmission rate is even faster in crowded spaces with limited ventilation [284]. Since its emergence, there have been over 422 million confirmed cases and above 5.8 million deaths due to COVID-19 worldwide [286]. Although most cases occur in adults with underlying comorbidities such as diabetes, cardiovascular diseases, and chronic pulmonary disorders, COVID-19 is also known to occur in pediatrics with 1–5 % of confirmed cases accounting for children below the age of nineteen-year-old [287,288,289,290,291,292,293,294]. Children, however, often display mild forms of the disease, have a better prognosis, and thus a lower mortality rate from the virus as compared to adults infected with COVID-19 [289,292,295,296]. Some studies have also been undertaken to determine the disease’s effect on pregnancy and neonates, and the findings obtained suggests that pregnant women are more likely to develop severe forms of COVID-19 than non-pregnant women [295,297,298,299]. 

The coronavirus disease is a global emergency and possibly a nightmare to healthcare systems due to the overload it has imposed on global healthcare facilities and personnel, which has been so much such that even well-developed countries were on the brink of a breakdown. With the way that COVID-19 prevalence and hospital admissions have overloaded the healthcare systems, it has negatively affected the control over other threats to global health such as TB, HIV/AIDS, and malaria [12,300,301,302]]. Laboratories are prioritizing COVID-19 diagnostic tests over many other important investigative tests for these infectious diseases [300]. As has already been established, TB is a poverty-related disease; thus, the emergence of a lockdown and the need to quarantine has had even more devastating effects, particularly in low-income countries. Many people have lost their jobs and as a result, they cannot afford to prioritize being able to cover the cost of traveling to health care centers for their TB treatment [303]. Some countries such as Sierra Leone have resorted to providing their TB patients with enough medication to last them a long while into the lockdown/quarantine periods. Though this is a good initiative, however, the absence of direct treatment monitoring through the DOTS program in such settings and difficult times does not assure proper adherence to treatment and could lead to increasing rates of therapy failure, development of drug resistance, and increased spread of TB infection [303], as well as a superinfection caused by the coronavirus in these populations [283]. Patients with existing latent TB infection and those with active TB are at a greater risk of contracting COVID-19 infection and are more likely to develop severe forms of the disease because of the lung damage already caused by the *Mtb* infection [283,300].

Children are faced with the challenge of under-nutrition as many schools closed because of repeated or intermittent lockdowns; thus, they no longer have access to feeding schemes [304,305,306,307]. This is deeply concerning for pediatric patients in the context of TB transmission because malnutrition is a predisposing factor to children contracting TB. The WHO estimated that over 1.1 million children under the age of fifteen contracted TB in 2018 and there were over an estimated 233,000 fatalities due to the disease in the same year [6]. The COVID-19 pandemic has caused the disruption of many TB prevention and control strategies such as BCG immunization, community case finding, contact tracing, and DOTs across the world [307]. China, for instance, has recorded a 36–52% decrease in TB notification rates (adults and children) since the lockdown as compared to the TB notification rates in the years 2015–2019 ([300]. This indicates that fewer people are seeking healthcare for TB-related symptoms, possibly due to the stigma of COVID-19, which increases the burden on the already existing stigma associated with TB [284,308,309,310,311]. Furthermore, possible confusion and delays in the diagnosis of pulmonary TB (the most prevalent form of TB infection) may have resulted from similarities in its signs and symptoms relative to the coronavirus disease, as well as limited access to healthcare facilities during the pandemic. The frequent lockdowns, particularly the stricter movement restrictions imposed at the early stages of the pandemic, favored close coexistence (increasing household spread from already diagnosed adult TB patients), and resulted in an increase in latent and active TB infection in pediatrics [2,312,313,314,315].

Overall, the coronavirus disease has significantly distorted TB treatment, prevention, diagnosis, and care for affected persons—adults, adolescents, and children alike [2]. All gains and progress made with TB prevention, diagnosis, treatment, and global eradication goals are now at risk because the coronavirus disease has taken over the center stages of healthcare platforms. This virus has not only increased the risk to people with TB but has led to serious disruptions to service delivery. The most conspicuous challenge posed has been the huge global drop in the number of newly diagnosed and reported TB cases. This reduced from 7.1 million documented in 2019 to 5.8 million recorded for 2020, indicating an 18% decline, back to numbers reported in 2012, and much lower than the approximately 10 million people who developed active TB disease in 2020. Increases in TB deaths associated with limited access to treatment and diagnosis have been observed for the first time in over one decade. In other words, the ongoing pandemic has triggered a reversal of years of progress made with making key TB services available and decreasing disease burden with many planned global targets being mostly off-track. The steady decline in the number of adults and pediatrics developing TB annually, which was accomplished in past years, has almost slowed down to a complete halt at this point. These unpleasant occurrences are predicted to be noticeably worse in the coming years [2]. Studies and forecasts reported to date indicate that if TB is not equally prioritized during this COVID-19 season, the TB morbidity and mortality rates within all age groups could be disastrous in the future. Thus, thorough evaluations of lessons learned, and the development of necessary actions could create avenues for improving TB management and alleviating current setbacks to ensure that established and upcoming TB programs are successfully executed despite the ongoing pandemic [2,6,312,313,314,315]. 

## 8. Pediatric Tuberculosis: Still a Global Challenge or Breakthrough?

Statistically, children represent the lower percentage of total TB cases worldwide. This may even be one of the reasons why not much focus has been on addressing the burden of TB in this specialized population. However, the occurrence of active TB disease in children is a direct reflection of the continuous *Mtb* transmission from infected adults. This is because minors progress to active disease and even more severe forms of this ailment soon after their initial infection. Moreover, children are at a higher risk of being carriers of latent TB infections that may become transmittable at a later stage. Thus, minors play a major role in infection spread across all age bands making the eradication or at least optimal control of active TB disease within the pediatric population extremely important. 

Although much progress has been made to improve the prevention, treatment, diagnosis, and overall management of the disease, more of this work has been undertaken to address these aspects in adults, and not so much for the pediatric population. The traditional reasons for this are the fact that the disease is paucibacillary in minors and that the symptoms of the disease often overlap those of other illnesses, making it difficult to diagnose TB in this patient category. Another age-old reason for not including children in pharmacokinetic studies for treatment and prevention purposes is that their biology and physiology change significantly as they grow older. Furthermore, compliance to medication regimens is difficult to achieve in this age group because the pill burden is high, the medicine is not palatable, and there are not enough child-friendly formulations available. The stumbling block to the development of child-friendly dosage forms has been that pediatric patients are sensitive to many excipients that could be used to improve palatability; these are excluded from clinical trials and therefore developing a totally new dosage form, introducing new pharmaceutical molecules, or even changing routes of administration would require clinical studies involving children, which could be ethically challenging to achieve. These reasons are true but should not be a hindrance, especially because there exist extensive data on how the titration of adult treatment solutions to children does not fully serve the best needs of children’s treatment outcomes. 

Even though scientists and stakeholders continue to do phenomenal work to address the crises caused by the relatively high incidence of TB in children and adolescents, there is so much more that still needs to be dealt with in the areas of optimizing diagnosis, pharmacotherapy, and prophylactic measures. For instance, the “end TB strategy” has been in place for over eight years, yet the number of minors being actively infected and dying from TB increases each year, a standardized way of accurately diagnosing TB in children is yet to exist, and the challenge to improve compliance to TB medications regimens in pediatric patients is yet to be addressed to full capacity—these are only a few of the examples of where the gap lies. Furthermore, novel technologies (e.g., the use of immunotherapeutic agents, diagnostic biomarkers, drug combinations, etc.) that are useful for pediatric-specific detection and pharmacotherapy of TB are underway, but these still have a long way to go when it comes to the actual implementation of these biotechnologies. Unfortunately, TB is a poverty-related ailment that mostly affects underdeveloped and/or developing communities, so the question is—will these advances be practical for this population in those settings? In our opinion, pediatric TB remains a global challenge affecting the well-being of many children and adolescents, and further advancements that would significantly contribute towards effective management that can improve the quality of lives and expectancies of affected minors or those at risk are very much needed. 

## 9. Conclusions

In our review, compiling up-to-date information on the management of TB in children and adolescents, we note that pediatric TB remains a substantial threat to the health and well-being of this specialized population globally. With an estimated annual burden of one million plus cases worldwide and a significant number remaining undiagnosed, TB infection within the pediatric population requires a closer look encompassing a concurrent multidisciplinary approach. From this review, we can outline that the main limitations to successful management of TB in children fall within the following areas: prevention, diagnosis, and therapeutic management. Successful prevention of TB in children is limited by the fact that there is a vast array of preventative treatment options; however, there is no optimum choice identified. The selection of the preventative treatment option to be used greatly depends on the discretion of the health care provider, which is often influenced by the medication availability or most commonly, the lack of needed resources. Despite the success of the BCG vaccine over the years, new vaccine options are needed. This is because BCG vaccine protection against pulmonary TB infection decreases with age, it does not protect against disseminated forms of TB, and it is ineffective against reactivation of latent TB. Therefore, the BCG vaccine is not a guaranteed prophylaxis option for TB transmission. It is important that preventative options that are to be developed should cater to all pediatric groups, including HIV-positive children. Moreover, this vaccine may cause the BCG-disseminated disease when administered to children who are HIV-positive. This alone shows that minors continue to be marginalized by the advancements in biomedicine. Added to the challenges in preventing TB in pediatric patients, once infected, it is even more difficult to accurately diagnose the disease. This is due to the lack of standardized diagnostic tests that are more sensitive to the presentation of the disease in children. Furthermore, a key component to the successful management of TB in pediatric patients relies heavily on them completing the course of treatment. This is often limited by the fact that the children’s compliance relies heavily on their parents’ or caregivers’ dedication. The complexity of administering the anti-TB drugs to children, which is often by means of off-label use, causes caregivers and parents to lose motivation. Ultimately, this leads to incorrect dosing, food–drug interactions, a reduction in drug potency, impaired stability of the formulation, sub-optimal therapeutic efficacy, and the development of drug resistance due to treatment failure. Therefore, a multipronged strategy focusing on optimal treatment, prevention, and diagnosis as well as addressing other important risk factors such as poverty, limited health coverage, security, and poor nutrition is necessary to make further progress towards strengthening already existing effective interventions worldwide. Furthermore, the cumulative impact of the setbacks caused by the onslaught of the COVID-19 pandemic makes prioritizing these proposals critical for curtailing TB spread, morbidity, and mortality rates in pediatrics. Summarily, research activities need to be geared toward accomplishing these proposed goals to enable more targeted breakthroughs and progress toward zero pediatric TB fatalities.
